# Analysis of Unstable Plastic Flow in the Porous 316L Samples Manufactured with a Laser 3D Printer

**DOI:** 10.3390/ma16010014

**Published:** 2022-12-20

**Authors:** Nataliya Kazantseva, Yulia Koemets, Denis Davydov, Nina Vinogradova, Igor Ezhov

**Affiliations:** 1 Institute of Metal Physics, Ural Branch of Russian Academy of Sciences, 620108 Ekaterinburg, Russia; 2 Department of Heat Treatment and Physics of Metals, Ural Federal University Named after First President of Russia B.N. Yeltsin, 620002 Ekaterinburg, Russia

**Keywords:** 3D printing, deformation instability, twins, austenitic steel

## Abstract

The study of unstable plastic flow in porous steel 316L samples after compression deformation at room temperature with different strain rates was carried out. The samples were obtained from ASTM F3184 medical grade steel powder by digital metallurgy using a Renishaw AM 400 laser 3D printer. Serrations on the stress-strain curves and strain localization bends were found, which were associated with the Portevin-Le Chatelier effect and testified instability of the plastic flow of the material under the deformation process. Deformation twins were observed in the structure of deformed samples.

## 1. Introduction

The importance of studying the physical nature of the elastic-plastic transition is growing with the increasing requirements for reliability and strength of parts and products. In addition, it is important when the laws of deformation strengthen change during the deformation process, and it is necessary to find out the reasons for arriving at the macroscopic localization of deformation.

Due to good mechanical and physical properties, 316L (EN 1.4404) austenitic steel products are widely used in various industries: from medicine, oil- and gas- pipeline parts, and reactor engineering. 316L steel is a suitable material for biomedical applications due to its negative strain-rate sensitivity (the flow stress decreases with an increase in the rate of deformation) [1]. Austenitic 316L stainless steel is ideal for implants such as bones, acetabulum (one-half of an artificial hip joint), and patella replacement, as well as for screws, plates, and prostheses in dentistry and orthopedics. Biomedical titanium alloy Ti-6Al-4V and 316L steel have good biocompatibility and strength properties. However, 316L has better deformability and fracture resistance, and is cheaper than titanium alloy [2]. Austenitic corrosion resistant steels have low strength properties after standard heat treatment. The main strengthening mechanisms of 316L steel are solid solution strengthening and Hall-Petch strengthening (an increase in hardness associated with the reduction of average grain size) [3]. Both mechanisms should be considered at the post-treatment of the parts and products made on 316L steel.

The increase in strength in alloys and steel at determined strain rates and temperatures are usually related to dynamic strain aging (DSA). Dynamic strain aging (DSA) in low-carbon steel is commonly associated with the so-called Portevin-Le Chatelier (PLC) effect, which is visible under certain regimes of strain rate and temperatures on the stress–strain curves as serrated plastic flow [4]. In this case, on the sample surface macroscopic bands, which are the strain localization bands, with thicknesses of a few millimeters (the so-called PLC bands) may be observed. It should be noted that austenitic 316L steel material is susceptible to dynamic strain aging (DSA) in the temperature range of 200–500 °C, and with strain rates of 10^−4^–10^−2^ s^−1^ [3,5,6]. Dynamic strain aging (DSA) is known as a phenomenon related to unstable plastic flow. Serrated tensile stress–strain curves were found by Diepold et al. in both the selective laser melted sample and the conventional 316L steel sample deformed by compression with strain rate jumps conducted between 25 and 877 °C and at a low strain rate range of 10^−5^–10^−3^ s^−1^ [3]. Seong-Gu Hong and Soon-Bok Lee [6] observed the serrated stress–strain curves in a conventional 316L steel sample during the high temperature low-cycle fatigue deformation. They suggested that such an unstable plastic flow was associated with the dynamic strain aging (DSA) occurring in the temperature range of 250–600 °C and strain rate range of 10^−2^–10^−4^ s^−1^ [6]. Serrations were not found in stress-strain curves obtained at the room-temperature deformation. Smooth tensile curves were observed by Lv et al. in 316L conventional steel samples at the room temperature deformation with a strain rate of 10^−3^ s^−1^ [7]. It was found in [7] that the strain rate sensitivity could be negative once the DSA phenomenon had occurred. This fact was associated with the suppression of the dislocations and deformation twins caused by adiabatic heating at high strain rate deformation.

Additive digital technologies today allow for it to be possible to obtain products of any complex geometric shape, with minimal post-processing and a significant reduction in manufacturing time. At the same time, as numerous studies have shown, metal products manufactured using a 3D laser printer by laser power bed fusion (L-PBF) have significant differences in structure and properties (mechanical and physical) compared to metals and alloys obtained by conventional methods [8]. This provides the necessity of studying the processes of deformation and destruction of L-PBF metal samples and products. Of particular interest are the studies of porous samples, as the most promising for use in medicine.

The aim of the present work is to study the phenomenon of unstable plastic flow in porous steel 316L samples manufactured by 3D laser printing after room temperature compression with different strain rates.

## 2. Materials and Methods

Samples were obtained with a Renishaw AM 400 laser 3D printer (Renishaw Inc., Wotton-under-Edge, UK). To create a certain degree of porosity in the samples, the laser power was reduced by 25% from that recommended by the manufacturer. ASTM F3184 powder (316L steel) was used. The chemical composition of the powder is shown in Table 1.

The density (92%) of the obtained samples was determined by the Archimedes method [9]. Compression testing up to 30% was carried out with an Instron testing machine; the deformation strain rates were: 2 × 10^−2^ s^−1^, 3 × 10^−3^ s^−1^, and 8 × 10^−4^ s^−1^. The range of selected speeds was determined by the capabilities of the Instron testing machine (minimum, average, maximum). The average strain rate (3 × 10^−3^ s^−1^) corresponded to the usual strain rate for samples of this size. Sizes of the tested samples were selected in accordance with GOST 25.503-97 (ASTM E-9) requirements. A DRON-3 (X-ray diffractometer (Bourevestnik, JSC, St. Petersburg, Russia) with Cu ka radiation was used for phase analysis. The ZEISS Cross Beam AURIGA scanning electron microscope (SEM) (Carl Zeiss NTS, Oberkochen, Germany) equipped with an EBSD HKL Inca spectrometer used an Oxford Instruments Channel 5 analyzing system and Tecnai G2-30 Twin transmission electron microscope FEI, Hillsboro, OR, USA) for structural and crystallographic texture studies.

The strain hardening exponent (*n*) used to describe the work hardening behavior was calculated according to the following equation [7]:

$$n=\frac{d\left(ln\sigma_{0.2}\right)}{d\left(ln\dot{\varepsilon }\right)},$$

where $\sigma_{0.2}$ was the yield strength and $\dot{\varepsilon }$ was the strain rate. The strain rate sensitivity was calculated as the slope in a linear relationship between the yield strength and strain rate in double logarithmic coordinates.

The average work hardening rate (WHR) was calculated as follows [10]:

$$\mathrm{WHR}=\frac{\sigma_{5\%}-\sigma_{0.5\%}}{\varepsilon_{5\%}-\varepsilon_{0.5\%}}.$$

## 3. Results

**Figure 1:**
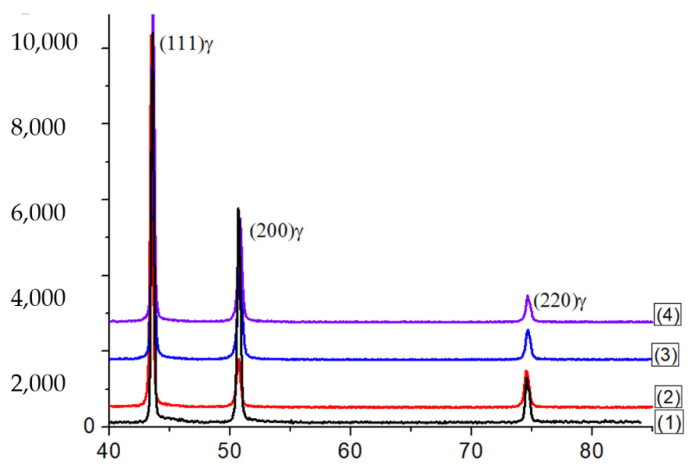
The X-ray diffractograms of the studied samples: (1) as-built; (2) 8 × 10^−4^ s^−1^; (3) 3 × 10^−3^ s^−1^; and (4) 2 × 10^−2^ s^−1^.

According to X-ray diffraction analysis, no changes in the phase composition were detected in the studied samples after deformation (Figure 1). The alloy samples had a phase composition of FCC austenite, both in the initial (L-PBF) state and after loading.

**Figure 2:**
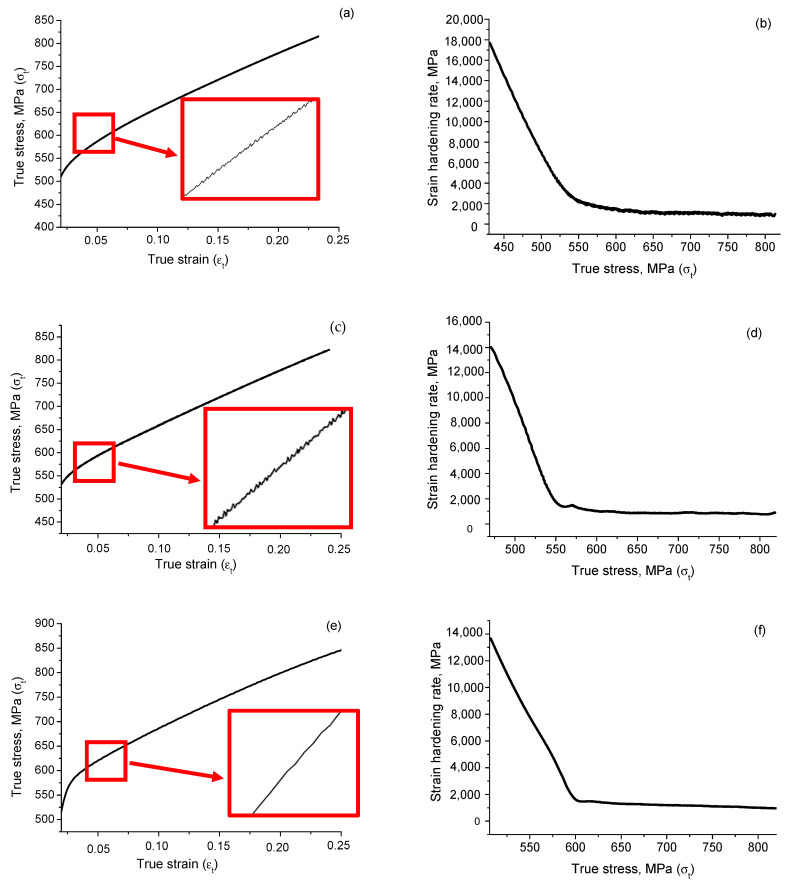
Results of the mechanical tests of the studied samples deformed at different strain rates: (**a**,**b**) 8 × 10^−4^ s^−1^; (**c**,**d**) 3 × 10^−3^ s^−1^; (**e**,**f**) 2 × 10^−2^ s^−1^.

Figure 2a,c,e shows the stress-strain curves of the studied samples. As demonstrated, the deformation process of the sample does not occur uniformly. After a certain level of plastic deformation, fluctuations in the deforming stress at the intermittent flow stage were noticeable on the loading curves obtained at strain rates 8 × 10^−4^ s^−1^ and 3 × 10^−3^ s^−1^. Stress drops reached 1–2 MPa with an increase in the percentage of deformation. The loading curve obtained at the highest strain rate 2 × 10^−2^ s^−1^ was observed to be smooth. The strain rate sensitivity in the studied strain range was calculated as positive *n* = 0.05. The results of the mechanical tests are presented in Table 2. The yield strength and the average work hardening rate (WHR) were found to be dependent on the strain rate and increased with the increasing of the strain rate.

The curves of the strain hardening rate $\theta =\frac{d\sigma }{d\varepsilon }$ versus the true stress (Kocks–Mecking plot) are presented in Figure 2b,d,f. Two sections with different lengths corresponding to the stages of linear deformation hardening, which associated with the different stages of microstructure evolution during deformation, may be observed in figures. Firstly, the strain hardening function exhibited a fast linearly decreasing, followed by a slowly decreasing with the increasing of the stress. Such behavior was usually observed in austenitic steel [11].

**Figure 3:**
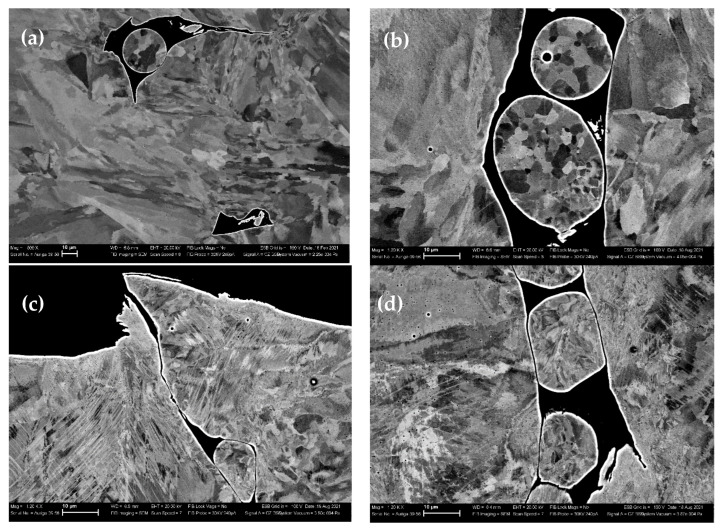
Porosity in the studied samples: (**a**) as-built; (**b**) 8 × 10^−4^ s^−1^, (**c**) 3 × 10^−3^ s^−1^, and (**d**) 2 × 10^−2^ s^−1^.

Figure 3 shows the data from the scanning electron microscopic examination (SEM) of the microstructure of the studied sample before and after loading with the different strain rates. Two types of pores were found in the initial (as-built) porous L-PBF sample, such as technological (irregularly shaped pores that could serve as stress concentrators and contributors to the destruction process during deformation) and gas pores (pores having a rounded shape). The shape of the technological pores was found to be transformed with increasing strain rate (Figure 3b,c). The technological pores in the samples deformed at strain rates 3 × 10^−3^ s^−1^ and 2 × 10^−2^ s^−1^ and were partially closed and the powders inside the pores were deformed. Rounded gas pores did not undergo any change. This fact correlated with the literature data, from which it was known that practically speaking, gas pores do not participate in the deformation process [12].

**Figure 4:**
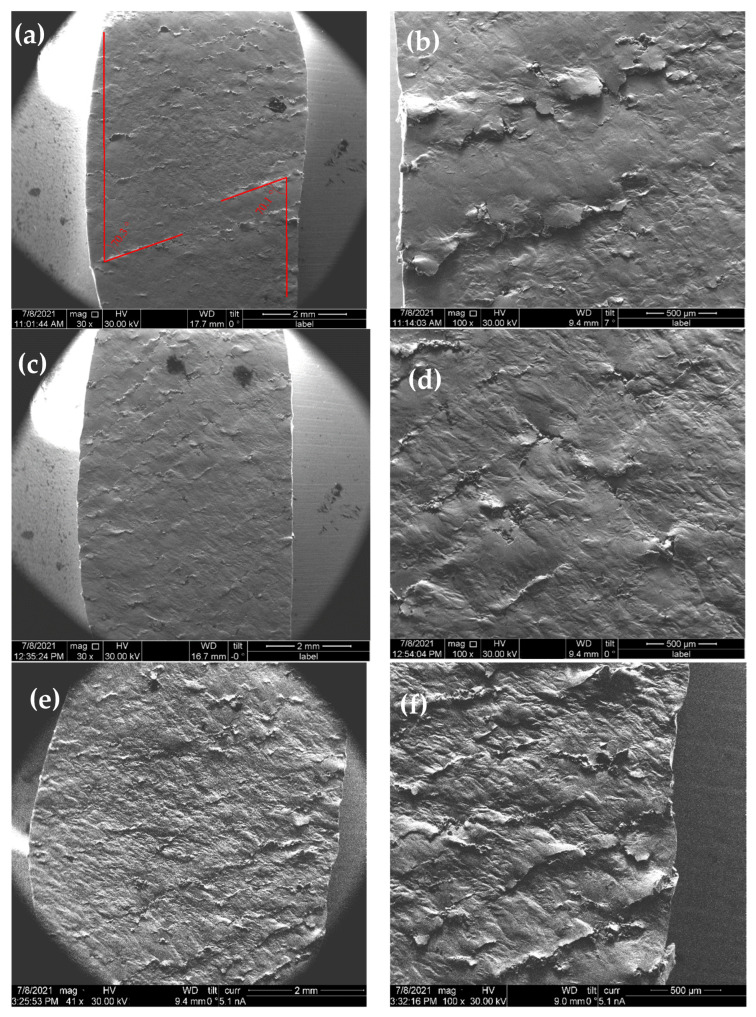
Strain localization bands in the studied samples after deformation, the different magnification, and scanning electron microscopy (SEM): (**a**,**b**) strain rate 2 × 10^−2^ s^−1^; (**c**,**d**) strain rate 3 × 10^−3^ s^−1^; (**e**,**f**) strain rate 8 × 10^−4^ s^−1^.

Figure 4 presents images of the side surfaces of the deformed samples. The side surfaces of the samples before deformation were polished. Strain localization bands, which confirmed the presence of the Portevin-Le Chatelier effect, may be seen on the sample sides. The angle between the direction of deformation and the direction of the strain localization bands was 70°, which corresponded to the angle between <111> directions in a cubic crystal lattice. The strain localization bands arose periodically and the distance between them was about 1 mm (Figure 4a,c,e). Figure 4b,d,f show the structure of the localized flow bands obtained with a large magnification. The technological pores in these samples had an active part in the deformation process. Under the action of a compressive force, technological pores closed. Figure 4b,d,f, taken with a large magnification, demonstrated the strain localization bands. In the figures, the displacement of the material layer in the bands is clearly shown.

**Figure 5:**
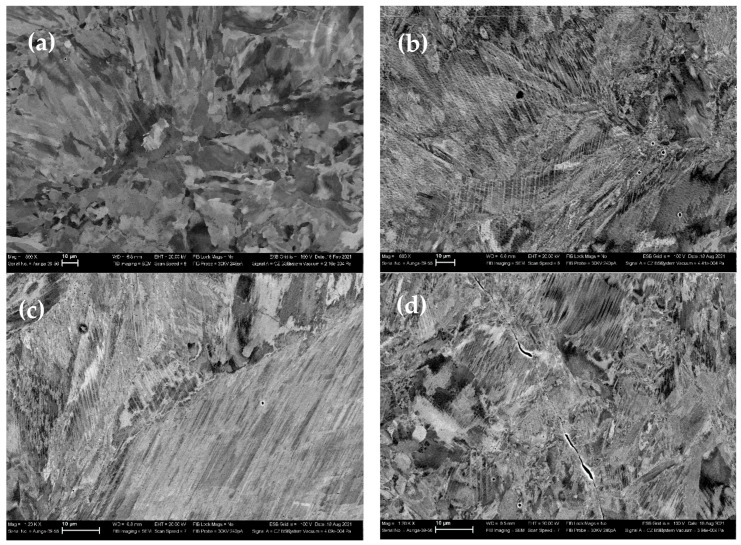
Microstructure of the studied samples, SEM images: (**a**) as-built; (**b**) 8 × 10^−4^ s^−1^, (**c**) 3 × 10^−3^ s^−1^, and (**d**) 2 × 10^−2^ s^−1^.

The cellular-dendritic structure was observed in the initial as-built sample; the grain sizes were determined to be from 10 to 40 microns (Figure 5a). In the deformed samples inside the grains, one can see the formation of elongated grains with an internal banded structure, which was associated with the process of twinning (Figure 5b–d).

**Figure 6:**
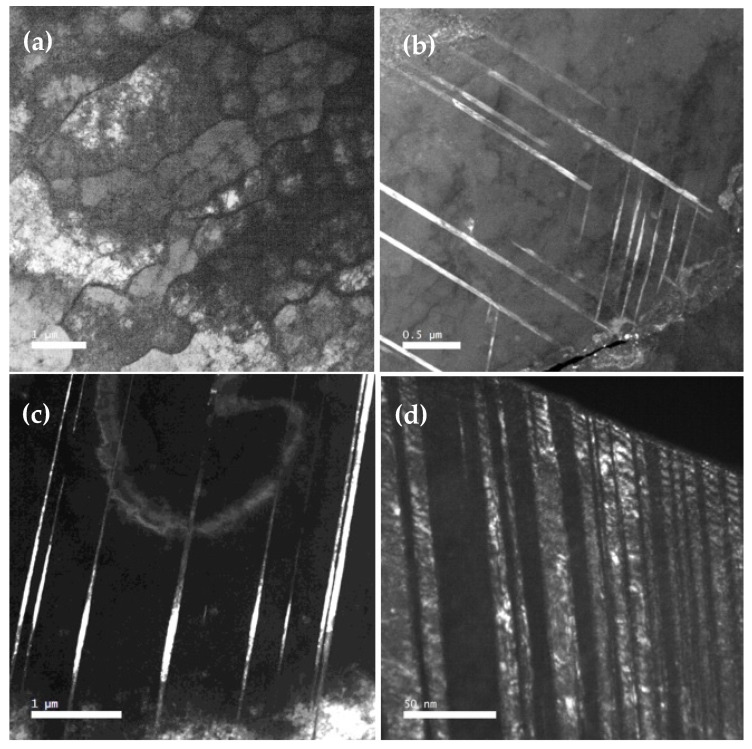
Microstructure of the studied samples, TEM images: (**a**) as-built; (**b**) 8 × 10^−4^ s^−1^, (**c**) 3 × 10^−3^ s^−1^, and (**d**) 2 × 10^−2^ s^−1^.

Figure 6 shows the transmission electron microscopic images of the microstructure of the studied samples. The cellular-dendritic structure of austenite with defect cell boundaries was observed in the as-built sample (Figure 6a). After the deformation by compression of up to 30%, the cellular-dendritic structure was preserved. The long twins running through the cell boundaries were found in the structure of the deformed samples (Figure 6b,c). FCC twinning with the simultaneous presence of two twinning planes of {111}-type twinning system was detected with SAED patterns taken from the twinning regions. In the samples deformed at strain rate 2 × 10^−2^ s^−1^, the twinning was changed by micro twinning (Figure 6d). Micro twins with width about of 10 nm were observed inside small regions. Figure 6d presents the dark-field image taken with twin reflex.

**Figure 7:**
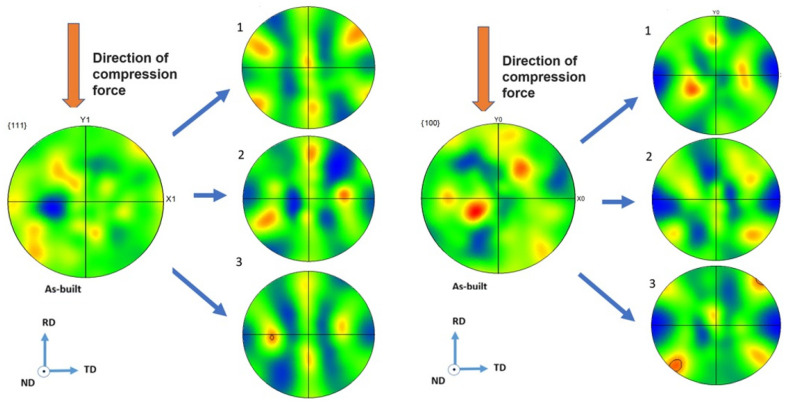
Pole figure with {100} and {111} zones obtained in studied samples after deformation with different strain rates, EBSD analysis: (1)—8 × 10^−4^ s^−1^, (2)—3 × 10^−3^ s^−1^, and (3)—2 × 10^−2^ s^−1^.

Figure 7 shows the results of an EBSD analysis of the studied samples. The initial as-built state may be related to the textureless sample. After deformation at a slow strain rate (8 × 10^−4^ s^−1^), the texture component (-1-12)[111], which was favorable to twinning, was observed. The increase in the strain rate led to an increase in the components Goss texture (01-1)[100] (Figure 7).

The deformation texture of FCC metals was determined, primarily, by the value of the stacking faults energy. However, in 3D printed 316L samples it was found that a sharp, cubic crystallographic texture {001}<100>, which depended on the technological process parameters of a 3D printer, determined the conditions of heat removal from the material under 3D printing [13]. The change of texture in 316L samples was also observed when the laser power was changed [8]. As was shown in [8], the crystallographic texture <011>, formed under an increase in the laser power and promoted the activation of deformation twins and nano-twins within the grains. In our case, the change in strain rate led to a change in crystallographic texture favorable to transit from twinning to micro twinning.

## 4. Discussion

According to the literature, dynamic strain aging (DSA) associated with the Portevin-Le Chatelier (PLC) effect is the thermally activated process determined by interaction between impure atoms and mobile dislocations [6]. The problem with the appearance of unstable plastic flow is of great practical importance, since the PLC effect plays a negative role in the processing of industrial alloys. Presents of the strain localization bands (PLC bands) decreases the mechanical properties of products and the plasticity of materials [14].

The appearance of serration on the loading curves, according to the literature data, is an indicator of the effect of the development of instability of the plastic flow or abrupt deformation of Portevin-Le Chatelier (PLC). Usually, the PLC effect manifests itself on material loading curves in the form of steps or teeth (serration) of various types that have a common appearance for different materials [5]. For each alloy, there is its own temperature-velocity region of existence of the PLC, where unstable plastic flow is not observed, and the deformation curves are smooth [14,15]. The process of twinning or martensitic transformation can be another variant of the manifestation of the PLC effect [16,17]. Plastic deformation by twinning has a localized character, due to which there is an abrupt nature of stress relaxation, causing the appearance of steps (serrations) on the deformation-stress curve. High temperature precipitations of the chromium carbides in steel materials can also cause the serration (fluctuations) on the stress-strain curves [5].

Particular austenitic stainless steels of the 300th grade may have a deformation unstable phase composition. In these steels, the formation of α’-phase deformation martensite can occur under effect of the external force and temperature. Austenitic steel 316L belongs to the type of room-temperature stable austenitic steels [18]. The formation of deformation martensite in 316L conventional austenitic steel, which leads to a decrease in plastic properties, was observed under low-temperature deformation or at high percentages of deformation (≥50%) at room temperature [19,20,21]. As for the DSA process associated with PLC effect in 316L conventional steel, the DSA process was observed at high temperature deformation. In this case, the strength of the sample increased, and the plasticity decreased [1].

In the case of the martensitic transformation, the strain-stress curves may also have serration and a change in the slope of the curve associated with the process of martensitic hardening. However, this effect is macroscopic and occurs with a significant percentage of martensite in the deformed sample [22].

The X-ray analysis in our study did not reveal the presence of diffraction reflexes of α′—martensite. This fact means that its volume fraction may have been less than 0.5 wt.%, which corresponded to the sensitivity limit of this method [23]. Being absent of the α′-phase reflexes also indicated that the observed oscillations (serrations) in the deformation curves were not associated with martensitic transformation. SEM and TEM studies supported the suggestion regarding the origin of the serration by twinning, rather than martensitic transformation.

The strain rate dependence of the mechanical properties observed in the present work also demonstrated the presence of the unstable plastic flow in the studied samples. It was known that mechanical properties of the materials were usually determined by tensile test. However, in the case of the study of the sample behavior under plastic deformation, the compression test was the most suitable, since it allowed for the occurrence of large deformations without the fracture of the specimen. It was found that the stress–strain curve of conventional materials in simple tension appeared the same as that in the case of simple compression at small strains. At large strains when the compression specimen was compacted and the tension specimen was in the plastic region, the tension and compression curves were different. This meant that in the region of small strains, the stress–strain curve of both compression and tension of the rigid or plastic material may be described by the Hollomon equation [24]:

$$\sigma =C\cdot \varepsilon^{n}.$$

where *C* is a constant stress and n is a strain-hardening exponent, usually lying between 0 and 1. When *n* = 0 the material is a perfect plastic, and when *n* = 1 the material is perfectly elastic-solid. The strain-hardening exponent was found dependent on the grain size, temperature, and strain rate. The value of the strain hardening exponent *n* = 0.05 was found by Singh et al. at room temperature deformation in the strain rate range 5 × 10^−3^ s^−1^–9 × 10^−2^ s^−1^ for conventional austenitic 316L steel with a grain size of 6 μm [24]. L-PBF samples in the present work had the grain sizes ranging from 10 to 40 μm and the range of strain rates was 10^−4^–10^−2^ s^−1^. This meant that in our study, hardening could not be explained by the change in grain size (Hall-Petch strengthening).

Portevin–Le Chatelier bands observed in the present work in L-PBF porous samples were unusual for conventional austenitic steel deformed at room temperature [25]. As we suggested, the value of critical strain, which was needed for the activation of the Portevin-Le Chatelier effect (PLC) was decreased in porous L-PBF samples due to the high level of defects.

## 5. Conclusions

The obtained results may be summarized as the following:

- A study of the deformation process in porous 316L steel samples showed that compression deformation of up to 30% at room temperature in the range of strain rates 10^−4^–10^−3^ s^−1^ contributes to unstable plastic flow described by the Portevin-Le Chatelier effect.
- Small teeth (serration) are formed on the strain-stress curves, indicating the heterogeneity of the flow of plastic deformation in the studied samples.
- The localization of plastic deformation at the stage of intermittent fluidity in the form of single deformation bands are due to the Portevin–Le Chatelier effect. Strain localization bands were found on the side surface of the deformed samples, and the direction of the bands corresponded to the crystallographic directions in the cubic lattice.
- Deformation twinning was found in the structure of all deformed samples.
- The technological pores, which are present in the as-built sample of 316L steel, take part in the deformation process and close under compressing force.

## Figures and Tables

**Table 1:** Chemical composition of studied alloys, wt.%.

Fe	C	Si	Mn	Ni	S	P	Cr	Mo	N
Bal.	≤0.03	≤0.75	≤2	10-14	≤0.03	≤0.045	16-18	2-3	≤0.1

**Table 2:** Results of mechanical tests.

Strain Rate	*σ*_0.05,_ MPa	*σ*_0.2,_ MPa	WHR
8 × 10^−4^ s^−1^	345	436	84
3 × 10^−3^ s^−1^	408	476	91
2 × 10^−2^ s^−1^	437	516	109

## Data Availability

Data are available by request.
